# GLT25D2 Is Critical for Inflammatory Immune Response to Promote Acetaminophen-Induced Hepatotoxicity by Autophagy Pathway

**DOI:** 10.3389/fphar.2020.01187

**Published:** 2020-09-18

**Authors:** Xiaohui Zhang, Lele Guo, Xiangying Zhang, Ling Xu, Yuan Tian, Zihao Fan, Hongshan Wei, Jing Zhang, Feng Ren

**Affiliations:** ^1^ Department of Hepatology, Beijing Youan Hospital, Capital Medical University, Beijing, China; ^2^ Beijing Institute of Hepatology, Beijing Youan Hospital, Capital Medical University, Beijing, China; ^3^ Department of Gastroenterology, Beijing Ditan Hospital, Capital Medical University, Beijing, China

**Keywords:** ****acetaminophen, acute liver injury, autophagy, GLT25D2, liver inflammation

## Abstract

Acetaminophen (APAP) overdose induces hepatocyte necrosis and causes liver hepatotoxicity. Currently, the role of galactosyltransferase in APAP-induced liver injury is still unclear. This study assessed the contribution of the GLT25D2 gene, a kind of collagen galactosyltransferase, to the development of APAP-induced liver injury. This study found that the expression of GLT25D2 markedly increased first and then decreased in the liver of mice treated with APAP, however, it downregulated in the liver of APAP overdose-patients compared with normal controls. Knockout of GLT25D2 significantly ameliorated the liver injury, meanwhile, it downregulated the proinflammatory cytokines (IL-6, TNF-α) and chemokines (CXCL-10, MIG and CXCL-1) levels, however, and upregulated the anti-inflammatory cytokines (IL-22, IL-10) levels. Mechanistic explorations showed that (1) GLT25D2 knockout promoted autophagy pathway; and (2) the GLT25D2 knockout-induced autophagy selected to clear damaged mitochondria in APAP-induced liver injury by mitophagy; and (3) the autophagy intervention by Atg 7 siRNA cancelled liver protection by knockout of GLT25D2 through regulating liver inflammation. In conclusion, our study proves that the upregulated expression of GLT25D2 decreased autophagy contributing to APAP-induced hepatotoxicity by mediating the inflammatory immune regulatory mechanism.

## Introduction

Acetaminophen overdose can cause liver failure, which is a major cause of death in the developed world (Kaplowitz, 2004; Lee, 2017). N-acetylcysteine (NAC) is a standardized treatment for patients with APAP overdose (Athuraliya and Jones, 2009; Bernal et al., 2015). Although the nature of hepatotoxicity induced by APAP overdose has been explored, the detailed mechanisms of liver injury are still not fully understood.

Protein glycosylation refers to the formation of glycosidic bonds with amino acid residues in proteins catalyzed by glycosyltransferases, which play a critical role in modifying proteins and exerting protein function. Collagens are important components of the extracellular matrix, and regulate many biological processes (Myllyharju and Kivirikko, 2004). The collagen galactosyltransferases, *GLT25D2* and *GLT25D1* genes, encode Hyl-specific galactosyltransferase enzymes and regulate collagen glycosylation (Schegg et al., 2009; Liefhebber et al., 2010; Baumann and Hennet, 2016). Despite the fact that GLT25D1 and GLT25D2 have recently been identified as glycosyltransferases, the study on the biological significance of GLT25D1 or GLT25D2 is lacking. Therefore, it is necessary to investigate the functions of GLT25D1 or GLT25D2 in various diseases.

Macroautophagy is an important physiological process that conserves the turnover of intracellular substances in eukaryotes. In this process, some damaged proteins or organelles are wrapped in autophagosome and then sent to lysosomes for degradation and recycling, thereby redistributing nutrients for the maintenance of cellular energetic balance (Yorimitsu and Klionsky, 2005). Moreover, the studies have been suggested that autophagy plays the critical role in cell differentiation, anti-microbial response, and death (Ohsumi, 2001; Levine and Deretic, 2007; Mizushima et al., 2008). Our previous studies have reported that autophagy protected mice from acute liver failure induced by lipopolysaccharide (LPS) and D-galactosamine (D-GalN) (Jiao et al., 2014; Ren et al., 2016). Autophagy also conferred protection against APAP-induced hepatotoxicity (Igusa et al., 2012; Ni et al., 2012; Ni et al., 2016).

Our previous study has demonstrated that GLT25D2 is a negative regulator of autophagy (Zhang et al., 2018), but its detailed function in APAP-induced hepatotoxicity is not clear, so this study aimed to investigate the critical role of GLT25D2 in mechanisms of APAP-induced hepatotoxicity, and further evaluate its potential pathophysiological relevance. It demonstrated that GLT25D2 is upregulated and further promotes hepatotoxicity induced by APAP overdose. Detailed mechanistic investigations provided evidence for a novel role for GLT25D2 in regulating liver inflammation mediated autophagy pathway in the pathogenesis of APAP-induced liver injury.

## Materials and Methods

### Generation of GLT25D2 Knockout Mice

Details are described in reference (Wei et al., 2013b). Briefly, the neomycin cassette served as the positive selection marker during the embryonic stem cell targeting step. It was eliminated through expressing Cre recombinase by a plasmid in the targeted ES cells. Partial exons 2 and total exons 3 were removed and replaced with loxpNeoloxp. The translation was stopped in exons 2 when meeting the stop code, which was added in-framely into exons 2. There is no difference observed from birth until more than 1 year between the characteristics of the GLT25D2^+/+^ and GLT25D2^-/-^ mice.

### Mice Experimental Protocol

Wild-type C57BL/6 mice and GLT25D2 knockout C57BL/6 mice were used according to the principles of the 3Rs (replacement, reduction, and refinement). All mice received humane care according to the Animal Care Committee guidelines of Capital Medical University. The animal protocol had been approved by the Institutional Animal Care & Use Committee (IACUC) of Capital Medical University. For *in vivo* studies, mice were administered either saline or acetaminophen (APAP, 500 mg/kg, i.p.). Autophagy was knocked down by injection of siRNA for Atg 7 (0.5 nmol/g) to mice 24 h before administering APAP. Chloroquine (CQ, 60 mg/kg) was administered to mice 2 h before APAP. Recombinant mouse IL-10 (rIL-10; 1μg/mouse) was administered to mice 2 h before APAP.

### APAP-Induced Hepatotoxicity Measurement

To assess liver damage, serum aspartate aminotransferase (AST) and alanine aminotransferase (ALT) were measured. Liver tissue was fixed with 10% neutral formaldehyde and embedded in paraffin. Sections (4μm) were cut and stained with hematoxylin and eosin, and then detected under a light microscope.

### Cyp2E1 Assay and Glutathione Assay

Cyp2E1 activity was measured using a p-nitrophenol assay and glutathione (GSH) was measured in liver homogenates as described in Steven R. McGreal and Bharat Bhushan (2018) and Wu D and Cederbaum A (2008) (Wu and Cederbaum, 2008; McGreal et al., 2018).

### Quantitative Polymerase Chain Reaction (qRT-PCR) Analysis

QRT-PCR was performed for mRNA expression of relative genes. RNA was extracted using TRIzol reagent and then reversed transcription into cDNA. Expression of the hypoxanthine-guanine phosphoribosyltransferase (HPRT) expression was used to standardize the samples. The mRNA levels used the 2–ΔΔCT method for calculation (Livak and Schmittgen, 2011).

### Immunoblot Analysis

Protein was extracted from samples in RIPA buffer together with phosphatase and protease inhibitors. Antibodies against GLT25D2 (Sigma-Aldrich, MO, USA), CYP2E1(Santa Cruz Biotechnology, TX, USA), β-actin, Atg7, p62, and LC3B (Cell Signaling Technology Inc., CA, USA) were selected. Finally, the quantitative results were assessed using the ImageJ software for comparisons between different groups.

### Plasma Cytokines Measurement

Cytokines of plasma were analyzed using ProcartaPlexTM Multiplex Immunoassay (eBioscience, Austria) including interleukin (IL)-1β, tumor necrosis factor (TNF)-α, IL-12(p70), IL-22, IL-10,IL-4, IL-5, IL-6, IL-12(p40), C-X-C motif chemokine(CXCL)-10, CXCL-1, monocyte chemotactic protein 1(MCP-1), monokine induced by IFN-γ (MIG), macrophage inflammatory protein-2(MIP-2), and RANTES.

### Transmission Electron Microscopy

An electron microscopy for detecting the number of autophagosomes was performed on liver samples from mice. For details on methods, please refers to (Zhang et al., 2017).

### Hepatocytes Experiments Protocol

Primary hepatocytes were isolated and cultured as described (Klaunig et al., 1981). For *in vitro* studies, the hepatocytes were given different doses of APAP at various time points. Autophagic markers were tested by LC3-II levels, p62 degradation, autophagosome numbers, and quantifying GFP-LC3 puncta numbers with or without the lysosomal inhibitor CQ. The GFP-LC3 plasmid infected hepatocytes within 24 h and/or incubated with Mito-Tracker Red (Invitrogen, CA, USA) for 0.5 hours so as to explore the effects of GLT25D2 on mitophagy.

### Knockdown of Atg7 by siRNA In Vivo

The siRNA was used to knockdown Atg7 (0.5nmol/g; Jima, Suzhou) to inhibit autophagy, the sequence was 5’-GCAUCAUCUUCGAAGUGAATT-3’.

siRNA using an Entranster *in vivo* transfection reagent (Engreen Biosystem Co, Beijing) was performed to knockdown Atg7 expression according to the manufacturer’s instructions.

### Human Specimens

Normal liver tissues from six subjects undergoing hepatic resection for hepatic cysts or for colorectal metastasis were collected. Liver samples of APAP-induced liver injury from eight patients undergoing liver puncture biopsy were obtained. Human specimens are used in this study which requires an informed consent from the patients. The protocol of this study was approved by the Medical Ethics Committee of Beijing YouAn Hospital, and met the ethical guidelines of the Declaration of Helsinki (1975).

### Immunohistochemistry Staining

After being treated according to normal procedure, the sections were incubated with the GLT25D2 rabbit antibody (1:1000; ab122192) at 4°C for 12 h, then incubated with Alexa Fluor 488 goat anti-rabbit IgG (A-11034, Invitrogen) for 45 min. DAPI (1 μg/mL; Shanghai, Shizebio) was used to stain the nuclei.

### Statistical Analysis

Quantitative data were expressed as the means ± SEM, which were subjected to unpaired Student t-test or single-factor analysis of variance. The probability value P ≤ 0.05 was considered significant.

## Results

### Dynamic Expression Profile of GLT25D2 in APAP-Induced Hepatotoxicity

**Figure 1:**
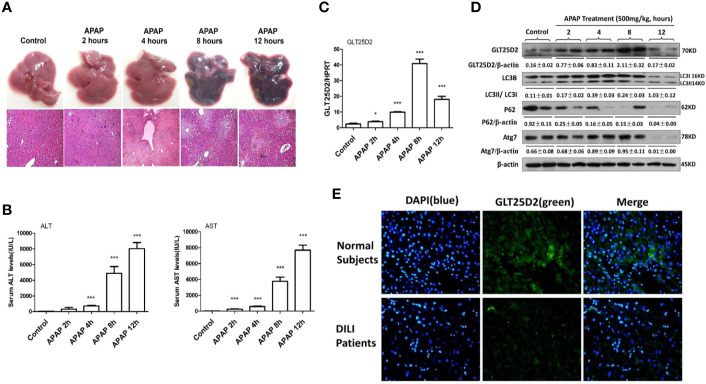
Dynamic expression profile of GLT25D2 and autophagy in the progression of APAP-induced hepatotoxicity. The mice were sacrificed after a single injection of APAP by intraperitoneal administration (500 mg/kg) at 2 h, 4 h, 8 h, and 12 h (16 mice/group, 8 males and 8 females). The mice in the control group (n = 16, 8 males and 8 females) were injected with saline only. **(A)** Representative livers and H&E staining (200×) of livers. **(B)** Serum AST and ALT enzyme levels. Compared with control group, ***P < 0.0001. **(C)** Gene expression of GLT25D2 was measured by qRT-PCR in the livers. Compared with control group, *P < 0.05, ***P < 0.0001. **(D)** Protein expression levels of GLT25D2, LC3B, p62, and Atg7 were evaluated by western blot assays in livers. A representative blot for three samples from each group is shown. Densitometry analysis of the proteins was performed for each sample. **(E)** Immunofluorescence staining for GLT25D2 (green) in the liver of normal subjects and patients with APAP overdose. Representative images of each experiment are shown. Original magnification, ×400.

The study first examined how GLT25D2 was regulated in APAP-induced hepatotoxicity. In response to APAP 500 mg/kg dose at different times, the changes of liver morphology, pathological lesions, and sALT and sAST levels were apparent after 8 h (**Figures 1A, B**). Hence, a mouse model for APAP-induced injury was successfully constructed at 8 h with a 500 mg/kg dose, which was used for subsequent experiments.

In addition to liver injury, the time-dependent APAP treatment significantly increased GLT25D2 expression in the liver before 8h, however, which was markedly downregulated in response to APAP treatment (500 mg/kg) for 12 h (**Figures 1C, D**). The protein levels of autophagic genes, such as LC3II conversion and Atg7, were increased after 2 h compared with normal mice, but they declined after 12 h in response to APAP 500 mg/kg treatment; the p62 protein degradation, known as a marker of autophagic flux (Jørkøy et al., 2009), progressively decreased responding to time-dependent APAP treatment (**Figure 1D**). Thus, the GLT25D2 and autophagy were promoted in the early stage of liver injury induced by APAP treatment in mice.

Using liver tissues of patients with APAP overdose, we measured the changes of the GLT25D2 protein. Unexpectedly, the results of immunofluorescence staining showed that the level of the GLT25D2 protein was lower in the liver of the patients compared with normal subjects (**Figure 1E**). Therefore, the protein levels of GLT25D2 was downregulated in the patients with APAP overdose.

### GLT25D2 Knockout Protects From APAP-Induced Liver Injury in Mice

**Figure 2:**
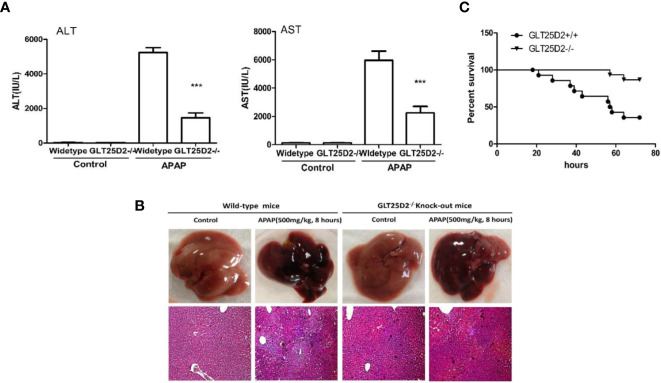
Knockout of GLT25D2 protected against APAP-induced hepatotoxicity. The wild-type and GLT25D2^–/–^ mice were administered with APAP (500 mg/kg, i.p.) or saline for 8 h (*n* = 16, 8 males and 8 females). **(A)** Serum AST and ALT levels from different groups. Compared with the APAP-induced wild-type mice group, ***P < 0.0001. **(B)** Representative livers and H&E staining (200×) of livers from different groups. **(C)** The survival rate of mice was measured in the GLT25D2^+/+^ mice and GLT25D2^–/–^ mice groups (15 mice/group).

Here, the role of GLT25D2 in APAP-induced hepatotoxicity was evaluated. Notably, the elevation of sALT levels, sAST levels, and the hepatic necrosis area induced by APAP treatment was substantially reduced in GLT25D2^–/–^ mice compared with wild-type mice (**Figures 2A, B**). Moreover, mortality was dramatically reduced in GLT25D2^–/–^ mice compared with GLT25D2^+/+^ mice, who were monitored for 72 h after APAP exposure (**Figure 2C**). Thus, these results suggested that GLT25D2 deficiency protects from liver injury and decreases mortality induced by APAP overdose in mice.

### Reduced Liver Inflammation in GLT25D2–/– Mice in Response to APAP

**Figure 3:**
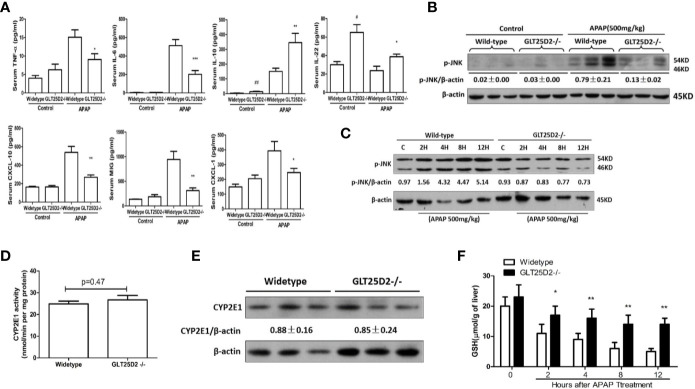
Reduced liver inflammation in GLT25D2^–/–^ mice in response to APAP. The wild-type and GLT25D2^–/–^ mice were administered with APAP (500 mg/kg, i.p.) or saline for 8 h (*n* = 16, 8 males and 8 females). **(A)** The plasma levels of TNF-α, IL-6, IL-10, IL-22, CXCL-10, CXCL-1, and MIG were measured using the ProcartaPlex. Compared with the APAP-induced wild-type mice group, *P <0.05, **P <0.01; ***P <0.0001. Compared with the wild-type mice control group, ^#^ *P* <0.05, ^##^ *P* <0.01. **(B)** Western blot analysis of the expression of the p-JNK and β-actin in livers. A representative blot for three samples from each group is shown. Densitometry analysis of the proteins was performed for each sample. **(C)** Western blot analysis of the expression of the p-JNK and β-actin in livers. A representative blot for one sample from each group is shown. Densitometry analysis of the proteins was performed for each sample. **(D)** p-Nitrophenol assay for CYP2E1 activity from isolated liver microsomes from wide-type mice and GLT25D2–/– mice. **(E)** Western blot analysis of the expression of the CYP2E1 and β-actin in livers. A representative blot for three samples from each group is shown. Densitometry analysis of the proteins was performed for each sample. **(F)** Hepatic GSH levels were measured in liver of mice treated with 500mg/kg APAP at 0 h, 2 h, 4 h, 8 h, and 12 h.

The impact of GLT25D2^–/–^ on the inflammation in mice was measured to investigate the protective mechanisms of GLT25D2 deficiency. Indeed, knockout of GLT25D2 significantly attenuated the levels of IL-6 and TNF-α in serum, which are proinflammatory cytokines, and decreased the serum levels of chemokines, including CXCL-1, MIG, and CXCL-10 in response to APAP stimulation. Interestingly, GLT25D2 knockout significantly augmented the levels of IL-10 and IL-22 in serum (**Figure 3A**). Moreover, knockout of GLT25D2 also suppressed phosphorylation of JNK (p- JNK) (**Figures 3B, C**), an important marker of APAP-induced toxicity (Henderson et al., 2007; Hanawa et al., 2008). Thus, these results suggested that the knockout of GLT25D2 significantly relieved liver inflammation, thereby protecting mice from APAP-induced hepatotoxicity.

To determine whether GLT25D2 deficiency affects the metabolic activation of APAP, we measured hepatic CYP2E1 activity, CYP2E1 protein expression, and the levels of hepatic glutathione (GSH). There was no difference in microsomal CYP2E1 activity and in CYP2E1 protein expression between WT and GLT25D2-deficient mice (**Figures 3D, E**). GSH is the main cellular antioxidant molecule that quenches NAPQI, the reactive metabolite of APAP, and scavenges mitochondrial ROS and peroxynitrite. Compared with wide-type mice, hepatic GSH levels were higher in GLT25D2-deficient mice at 4h, 8h, and 12 h (**Figure 3F**).

### GLT25D2 Knockout Promotes Autophagy in Responses to APAP

**Figure 4:**
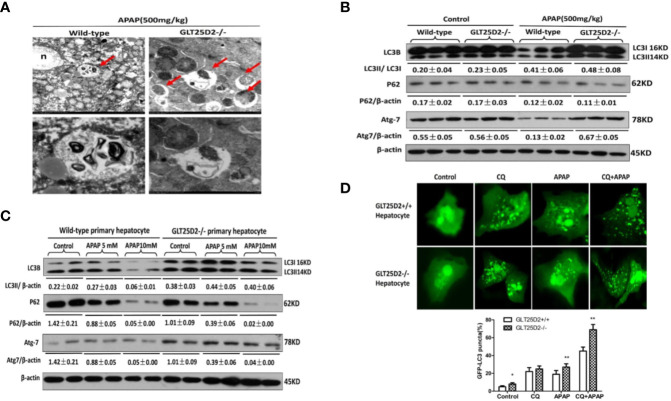
Knockout of GLT25D2 promoted autophagy responses to APAP *in vivo* and *in vitro*. The wild-type and GLT25D2^–/–^ mice were administered with APAP (500 mg/kg, i.p.) or saline for 8 h (*n* = 16, 8 males and 8 females). **(A)** Representative TEM images of liver samples from mice. Arrows indicate autophagosomes. **(B)** Western blot analysis of the expression of LC3B, p62, Atg-7, and β-actin in livers. A representative blot for three samples from each group is shown. Densitometry analysis of the proteins was performed for each sample. **(C)** The primary hepatocytes were incubated with APAP (5 or 10mM) for 12 h. The levels of LC3B, p62, Atg7, and β-actin were measured by western blotting. A representative blot for two samples from each group is shown. Densitometry analysis of the proteins was performed for each sample. **(D)** The primary hepatocytes were transfected with the GFP-LC3 plasmid for 24 h and pre-incubated with APAP (5mM) for 12 h to observe the formation of autophagosomes. GFP-positive cells were defined as cells that displayed bright, punctate staining (1000×). Approximately 50 cells were counted, and the experiment was repeated at least 3 times. Compared with the APAP-induced wild-type mice group, *P < 0.05, **P < 0.01.

Previous studies have suggested that the increased autophagy alleviated liver hepatotoxicity induced by APAP (Igusa et al., 2012; Ni et al., 2012; Ni et al., 2016). Our study has demonstrated that knockout of GLT25D2 can increase autophagy of liver in APAP-induced mice (Zhang et al., 2018). Here, we further explored the effects of GLT25D2 knockout on hepatic autophagy induced by APAP overdose. Transmission electron microscopy (TEM) analysis and western blotting results indicated the increased accumulation of autophagosomes and relative level of endogenous LC3II and Atg 7 compared with wild-type mice, but they did not significantly influence the degradation of p62 following APAP treatment in GLT25D2^–/–^ mice (**Figures 4A, B**). *In vitro*, the knockout of GLT25D2 increased the numbers of autophagosomes and the relative level of Atg7and LC3II, and promoted p62 degradation in primary cultured mouse hepatocytes induced by APAP treatment compared with primary cultured mouse hepatocytes from wild-type mice. Moreover, the number of APAP-induced autophagosomes per cell in GLT25D2^–/–^ hepatocytes was further increased in the presence of chloroquine (CQ), a kind of lysosomal inhibitor (Ni et al., 2011; Esteban-Martínez and Boya, 2015), compared with those in GLT25D2+/+ hepatocytes (**Figures 4C, D**). Thus, the results showed that the knockout of GLT25D2 promoted the autophagosome level during APAP-induced hepatotoxicity. Therefore, GLT25D2 might serve as a negative regulator of autophagy responses to APAP.

### Knockout of GLT25D2 Inhibits Liver Inflammation From APAP-Induced Hepatotoxicity Through Autophagy Pathway

**Figure 5:**
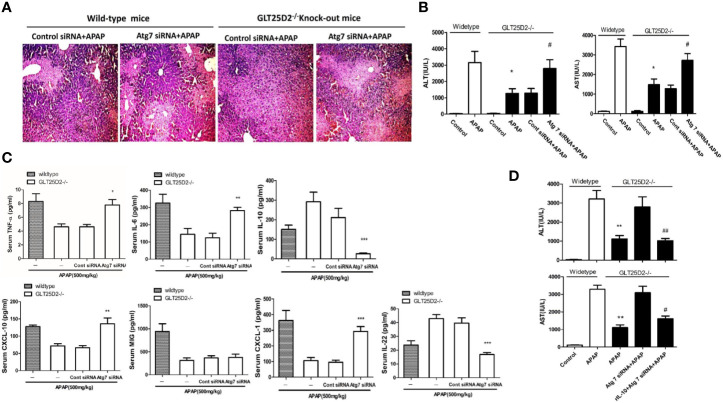
Knockout of GLT25D2 inhibits liver inflammation from APAP-induced hepatotoxicity through autophagy pathway. The mice were pretreated with Atg7 siRNA (0.5 nmol/kg) or control siRNA(0.5 nmol/kg) for 48 h *via* tail vein injection and then administered with APAP for 8 h (n = 10, 5 males and 5 females). **(A)** Representative H&E staining of livers (200×) from different groups. **(B)** Serum AST and ALT levels from different groups. Compared with the APAP-induced wild-type mice group, *P<0.05. Compared with the control siRNA and APAP-induced GLT25D2^-/-^ mice group, ^#^ *P*<0.05. **(C)** Serum levels of cytokines, including TNF-α, IL-6, IL-10, and IL-22, and chemokines, including CXCL-10, MIG, and CXCL-1. Compared with the control siRNA and APAP-induced GLT25D2^-/-^ mice group, *P<0.05, **P<0.01, ***P<0.0001. **(D)** The mice were pretreated with Atg7 siRNA (0.5 nmol/kg) or control siRNA(0.5 nmol/kg) for 48 h *via* tail vein injection, and then administered with rIL-10 protein (1μg/mouse, ip) 2 h before APAP treatment, then administered with APAP for 8 h (n = 10, 5 males and 5 females). Serum AST and ALT levels from different groups. Compared with the APAP-induced wild-type mice group, **P<0.01. Compared with the Atg 7siRNA and APAP-induced GLT25D2^-/-^ mice group, ^#^ *P*<0.05.

Next, the study sought to explore whether knockout of GLT25D2 protected the liver from injury *via* autophagy pathway. Our results demonstrated that hepatic protection by knockout of GLT25D2 in APAP-induced hepatotoxicity was partially negated by siRNA-induced knockdown of Atg7, which was proven by the relatively less preserved liver architecture on histological examination (**Figure 5A**) and the rebound levels of sALT and sAST (**Figure 5B**). Furthermore, knockdown of Atg7 siRNA again increased the serum levels of TNF-α, CXCL-10, IL-6, and CXCL-1 and again decreased IL-22 and IL-10 levels compared with those in GLT25D2^–/–^ mice treated with control siRNA and APAP (**Figure 5C**).

In order to further explore the direct relationship of autophagy and inflammation, we used the recombinant mouse IL-10 protein to treat APAP-induced mice on the basis of Atg7 siRNA intervention. The results showed that the treatment of the recombinant mouse IL-10 protein further reversed the effect of inhibiting autophagy on APAP-induced liver injury in GLT25D2^–/–^ mice (**Figure 5D**). Taken together, the inflammatory protective mechanisms of GLT25D2-knockout depended on autophagy in APAP-induced hepatotoxicity.

### Knockout GLT25D2 Promoted Mitophagy

**Figure 6:**
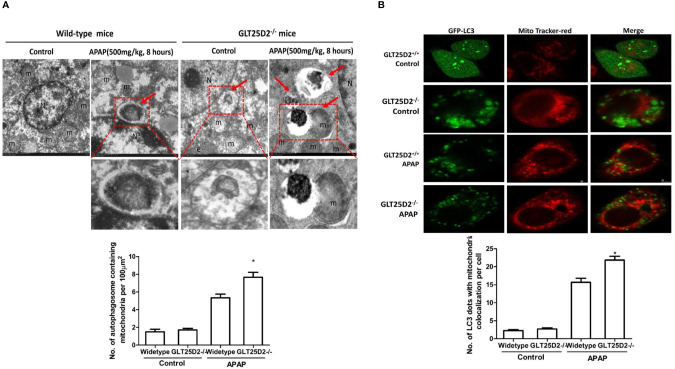
Knockout GLT25D2 promoted mitophagy. The wild-type and GLT25D2^–/–^ mice were administered with APAP (500 mg/kg, i.p.) or saline for 8 h (*n* = 16, 8 males and 8 females). **(A)** Representative liver TEM images of autophagosomes containing mitochondria are shown. Data (mean ± standard error of the mean (SEM)] were quantified. Arrows denote autophagosomes containing fragmented mitochondria. M, mitochondria; N, nuclei. Compared with the APAP-induced wild-type mice group, *P < 0.05. **(B)** The primary hepatocytes were transfected with the GFP-LC3 plasmid for 24 h and loaded with MitoTracker Red for 30 min before APAP (5mM) treatment for 12 h and examined by immunofluorescence analysis. The GFP-LC3 structures contained the mitochondria. Data were quantified (mean ± SEM, *n* = 3). Compared with APAP-induced GLT25D2^+/+^ hepatocytes, *P < 0.05.

Mitophagy, the selective engulfment and clearance of damaged mitochondria, plays an important role in the homeostasis of cell fate (Thomas and Gustafsson, 2013; Lemasters, 2014). TEM microscopy revealed that a notable fraction of damaged mitochondria was contained in autophagosomes in liver of APAP-treated wild-type mice, which was further enhanced in the liver of GLT25D2^–/–^ mice (**Figure 6A**). For the primary hepatocytes transfected with plasmid of GFP-LC3 and incubated with MitoTracker Red, APAP treatment significantly increased GFP-LC3 green dots, and some green dots were colocalized with the MitoTracker Red signals. The number of GFP-LC3 dots colocalized with MitoTracker Red further increased in GLT25D2^–/–^ hepatocytes (**Figure 6B**). Thus, knockout of GLT25D2 could protect mice from APAP-induced hepatotoxicity by promoting mitophagy.

## Discussion

**Figure 7:**
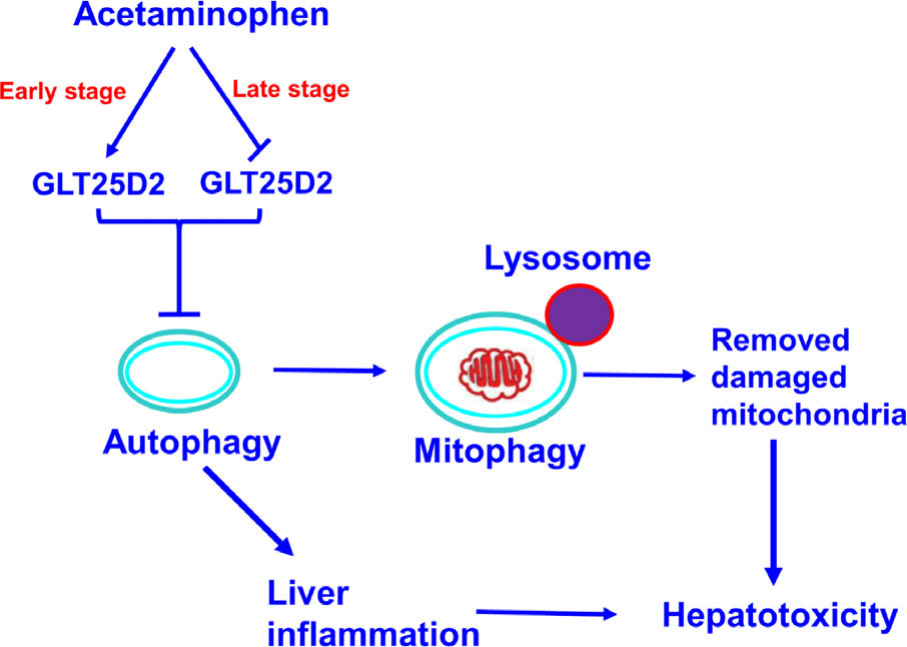
A proposed mechanisms model about the effects of GLT25D2 on APAP-induced liver injury. In the progression of APAP-induced acute liver injury, GLT25D2 was upregulated in the early stage and decreased in the late stage of APAP-induced acute liver injury, which promoted the downregulation of autophagy. These events resulted in promoting liver inflammation, and decreasing mitophagy, which attenuating the function of removing damaged mitochondria, ultimately inducing the development of APAP-induced hepatotoxicity.

The GLT25D1 and GLT25D2 proteins, two collagen (1-O) galactosyltransferases, have a strong galactosyltransferase activity toward collagen (Schegg et al., 2009). The physiological role of GLT25D2 in various diseases is still unclear. This novel study explored the pathological role in APAP overdose-induced liver injury using GLT25D2 knockout mice. It demonstrated that the collagen galactosyltransferase GLT25D2 level was firstly upregulated but later suppressed in the progression of APAP-induced liver injury. The increased GLT25D2 resulted in depressed autophagy, on the one hand, then to decrease mitophagy and further to promote APAP-induced hepatotoxicity; furthermore, to aggravate liver inflammation, and further to promote APAP-induced hepatotoxicity. Hence, the GLT25D2 autophagy pathway may be a critical mechanism to regulating liver inflammatory in APAP-induced hepatotoxicity (as depicted in **Figure 7**).

It is one important finding about the characteristic expression profiles of GLT25D2 in APAP-induced hepatotoxicity. The data demonstrated that the expression of GLT25D2 had a different trend in time-dependent APAP-induced hepatotoxicity in mice and patients with APAP overdose. The level of GLT25D2 was upregulated in the early stage (from 2 h to 8 h), but downregulated in the late stage of hepatotoxicity (after 8 h). However, the level of GLT25D2 significant decreased in the liver tissue of patients with APAP overdose. The reason for this difference was that many hepatocytes underwent necrosis in the late stage of APAP-induced hepatotoxicity in mice, leading to the reduction GLT25D2 synthesis or the degradation of GLT25D2. The patients with APAP overdose were hospitalized because of liver failure which occurred at the late stage of liver injury. The aforementioned results concluded that the expression of GLT25D2 was firstly upregulated and then downregulated with the progression of liver injury induced by APAP. The characteristic expression of GLT25D2 demonstrated that GLT25D2 might be one of the critical factors in the pathogenesis of APAP-induced hepatotoxicity.

Another important finding of this study was that the glycosyltransferase GLT25D2 shows the effect of promoting damage in APAP-induced hepatotoxicity by regulating liver inflammation. Glycosyltransferases transfer sugar to the acceptor molecules and are involved in many biological and pathological processes (Chang et al., 2011). Recent studies showed that some glycosyltransferases influence the occurrence and progression of liver disease. For example, the T-synthase (a glycosyltransferase) gene protected mice from fatty liver disease (Fu et al., 2008); the formation of bisecting-GlcNAc lead to the generation of fatty liver by disrupting the function of apolipoprotein B (Ihara et al., 1998); Glycosyltransferase GLT8D2 regulates ApoB100 protein expression in hepatocytes and participates in NAFLD pathogenesis (Wei et al., 2013a; Zhan et al., 2015). Only one study showed that glycosyltransferase GLT25D2 gene knockout promoted hepatocyte proliferation to a certain degree and inhibited hepatocyte apoptosis during liver regeneration (Wang et al., 2015). Sterile inflammation and innate immunity play an important role in the mechanisms of acetaminophen hepatotoxicity and repair (Hartmut et al., 2012). In our paper, the data showed that the knockout of GLT25D2 significantly protected mice from APAP overdose-induced hepatotoxicity and mortality, meanwhile, upregulated the expression of IL-10 and IL-22, and downregulated the expression of pro-inflammatory cytokines. The studies have shown that IL-10 and IL-22 both protect against acetaminophen-induced liver injury and lethality (Bourdi et al., 2002; Scheiermann et al., 2013; Feng et al., 2014), so we think that GLT25D2 takes part in the mechanism of APAP-induced hepatotoxicity by regulating IL-10 and IL-22. Moreover, our paper showed that GLT25D2 knockout promotes an autophagy response to APAP, and inhibition autophagy by using Atg7 siRNA again increased the serum levels of TNF-α, CXCL-10, IL-6, and CXCL-1 and again decreased IL-22 and IL-10 levels compared with those in GLT25D2^–/–^ mice treated with control siRNA and APAP (**Figure 5C**). Furthermore, the other study has shown that enhanced autophagy contributes to the protective effects of IL-22 against acetaminophen-induced liver injury (Mo et al., 2018). Above all, we take a hypothesis that knockdown of GLT25D2 protects from liver injury by regulating IL-10 and IL-22 mediated by autophagy pathway. This hypothesis need to be further explored.

This study also found that the regulation of mitophagy was one of the key mechanisms for GLT25D2 knockout in protecting against APAP-induced liver hepatotoxicity. Mitochondrial dysfunction was one of the important mechanisms of APAP-induced hepatotoxicity. APAP was transformed into NAPQI leading to mitochondrial oxidant stress, and further leading to JNK phosphorylation, which translocates to mitochondria triggering the oxidant stress (Jaeschke et al., 2012). Mitophagy is essential for normal cellular homeostasis and mitochondrial health, which is a selective form of autophagy and represents a critical mechanism controlling cell injury. It is well known that APAP-induced autophagy removes damaged mitochondria by mitophagy (Youle and Narendra, 2011; Ni et al., 2012). How APAP regulates autophagy and mitophagy is still unknown. Our results showed that APAP induced the increased GLT25D2 and autophagy in the early stage of liver injury, and knockout of GLT25D2 further promoted autophagy and mitophagy. Importantly, inhibition of autophagy reversed the protective role of GLT25D2 knockout in APAP-induced mice. So, the study demonstrated that the glycosyltransferase GLT25D2 was an important regulator of APAP-induced autophagy and mitophagy.

Moreover, we showed evidence that Cyp2e1 protein expression and enzyme activity does not significantly differ between WT and the GLT25D2 knockout mice (**Figures 3D, E**), however, the GSH levels seem to be higher in the knockout mice and are then significantly higher between 2 h and 12 h after APAP (**Figure 3F**). As GSH takes more than 4 h to metabolize a dose of 500 mg/kg APAP, the fact that GSH levels in knockout mice are significantly higher at 2 h and 4 h suggests that there is reduced NAPQI formation in the knockout mice. These may be due to change in p450 activities or changes in metabolism, i.e., increased glucuronidation and/or sulfation.

In summary, the present study provided new mechanisms for APAP-induced hepatotoxicity, that the GLT25D2 glycosyltransferase takes part in liver injury, partially regulating the inflammatory immune response mediated by autophagy pathway. Therefore, the intervention of GLT25D2 represents a new strategy to treat APAP overdose.

## Data Availability Statement

All datasets generated for this study are included in the article/supplementary material.

## Ethics Statement

All the animal protocols were reviewed and approved by the intramural Ethics Committee on Humane Treatment of Experimental Animals. Human specimens are used in this study which required a written informed consent from the patients. The protocol of this study was approved by the Medical Ethics Committee of Beijing YouAn Hospital.

## Author Contributions 

FR, HW, and JZ designed the paper. XHZ, LG, XYZ, and LX performed and analyzed experiments and wrote the paper. YT and ZF analyzed data and reviewed the paper. FR designed, supervised, and analyzed experimental work and wrote the paper.

## Funding

This study was supported by grants from the National Natural Science Foundation of China (81770611), demonstrating application and research of the Clinical Diagnosis and Treatment Technology in Beijing (Z191100006619096, Z191100006619097); Key Projects of Beijing Municipal Education Commission’s Science and Technology Plan (KZ202010025035) and the National Science and Technology Key Project on “Major Infectious Diseases such as HIV/AIDS, Viral Hepatitis Prevention and Treatment” (2018ZX10301407-005-002, 2018ZX10302205-004-004).

## Conflict of Interest

The authors declare that the research was conducted in the absence of any commercial or financial relationships that could be construed as a potential conflict of interest.
